# Recommending Unfunded Innovative Cancer Therapies: Ethical vs. Clinical Perspectives among Oncologists on a Public Healthcare System—A Mixed-Methods Study

**DOI:** 10.3390/curroncol28040254

**Published:** 2021-08-02

**Authors:** Osnat Bashkin, Keren Dopelt, Noam Asna, Nadav Davidovitch

**Affiliations:** 1 Department of Public Health, Ashkelon Academic College, Ashkelon 78211, Israel; dopelt@bgu.ac.il; 2 Department of Health Policy and Management, School of Public Health, Faculty of Health Sciences, Ben Gurion University of the Negev, Beer Sheva 8410501, Israel; nadavd@bgu.ac.il; 3 Oncology Institute, Ziv Medical Center, Safed 13100, Israel; noama@ziv.gov.il

**Keywords:** unfunded cancer therapies, ethical dilemma, medical cost-benefit

## Abstract

Over the past decade, there has been a growing development of innovative technologies to treat cancer. Many of these technologies are expensive and not funded by health funds. The present study examined physicians’ perceptions of the ethical and clinical aspects of the recommendation and use of unfunded technologies for cancer treatment. This mixed-methods study surveyed 127 oncologists regarding their perceptions toward using unfunded innovative cancer treatment technologies, followed by in-depth interviews with 16 oncologists. Most respondents believed that patients should be offered all treatment alternatives, regardless of their financial situation. However, 59% indicated that they often face dilemmas regarding recommending new unfunded treatments to patients with financial difficulties and without private health insurance. Over a third (38%) stated that they felt uncomfortable discussing the cost of treatment with patients. A predictive model found that physicians facing patients whose medical condition worsened due to an inability to access new treatments, and who expressed the opinion that physicians can assist in locating funding for patients who cannot afford treatments, were more likely to recommend unfunded innovative therapies to patients (F = 5.22, R^2^ = 0.15, *p* < 0.001). Subsequent in-depth interviews revealed four key themes: economic considerations in choosing therapy, patient–physician communication, the public healthcare fund, and discussion of treatment costs. Physicians feel a professional commitment to offer patients the best medical care and a moral duty to discuss costs and minimize patients’ financial difficulty. There is a need for careful and balanced use of innovative life-prolonging technologies while putting patients at the center of discourse on this complex and controversial issue. It is essential to develop a psychosocial support program for physicians and patients dealing with ethical and psychosocial dilemmas and to set guidelines for oncologists to conduct a comprehensive and collaborative physician–patient discourse regarding all aspects of treatment.

## 1. Background

With the rapid development of new and expensive medications and innovative treatment methods, objective considerations of innovative cancer treatments have become essential [1,2]. The value of a new treatment is usually determined by weighing the clinical benefit it provides against its cost [3]. Potential clinical benefit is usually defined by a treatment’s ability to prolong or improve patients’ lives. This is measured by examining the effect of the therapy on overall survival and its ability to maintain quality of life and control disease-related symptoms [4]. A study that analyzed 23 indicators of immunotherapy found that only three of them met the criteria of improved patient survival over time, and that data were not yet available regarding patient survival for 13 of the indicators due to their accelerated approval procedure in the U.S. Food and Drug Administration (FDA) [5]. Nevertheless, many of these expensive treatments are offered by oncologists. While some are funded by public healthcare systems or private health insurance companies, others are not.

Oncologists agree that there is a need for careful and balanced use of innovative technologies while putting the patient at the center of the decision-making process [6]. This balance requires meaningful interaction between a physician with clinical knowledge and patients who know their personal preferences, socioeconomic status, experiences, expectations, and degree of support from family and their immediate environment [7]. For patients in advanced stages of the disease, the challenge of meeting cost-benefit standards becomes impossible, as the value of life-years is not linear [8]. In these cases, which are the majority of those using the newest and most expensive cancer treatments, the personal and social value of the treatment is higher than its quantitatively measured and assessed utilitarian value. In addition, cost-benefit considerations may contravene ethical and social considerations, such as concern for equality in availability of medical care. Therefore, in decisions regarding expensive treatments, failure to achieve practical value may sometimes be justified by ethical considerations [9]. 

Despite medical guidelines for cooperative physician–patient discussions regarding the costs and benefits of innovative cancer treatments, the goals of such a discussion and how and when to conduct it are not adequately clear [10]. Some argue that oncologists have a social commitment to make careful and optimal use of limited resources [11]. Others argue that oncologists should be committed exclusively to the patient and make decisions based on the cost-benefit considerations of each case [12]. Another approach argues that it is not the role of oncologists to discuss financial issues and that the decision to offer appropriate treatment to the patient should be based solely on considerations of clinical efficacy [10]. As a result, although many physicians recognize the importance of having discussions with patients about clinical efficacy (which is often unclear) and the cost of treatment, few physicians conduct these discussions, presumably due to a lack of consensus on how and when to do so.

Under the National Health Insurance Law, Israel has a public healthcare system in which the state funds a designated set of medical treatments (basket of services) for every citizen. The funded treatments are updated every year, and new treatments and technologies are added. The Ministry of Health’s guidelines in Israel state that physicians should offer all treatment alternatives to the patient. The current study addresses the perceptions of oncologists regarding the use of unfunded treatments and technologies and the dilemmas that accompany the decision to do so. 

## 2. Methods

The study was approved by the Ashkelon Academic College Ethics Committee (Approval # 4-2019). Methods included a survey and in-depth interviews among oncologists.

### 2.1. Population Sample

Out of approximately 250 physicians specializing in oncology and members of the Israeli Society for Clinical Oncology and Radiation Therapy contacted by researchers, 127 responded to a telephone survey (50.8% response rate) between July and November 2020. The survey examined perceptions toward the use of new unfunded treatments. At the end of the survey, respondents were asked if they would be willing to be interviewed on the subject. Sixteen oncologists answered affirmatively, and interviews were conducted during the period of January to April 2021, after informed consent was obtained. Eight interviewees were male, and eight were female. Eight worked in two hospitals in southern Israel, seven worked in two hospitals in the center of the country, and one worked in a hospital in the north. Six were interns toward the end of their internship, and ten were specialist physicians. In addition, six of the respondents were current or former members of the ”Basket Committee”, which assesses the public funding of new treatments in Israel.

The interviewees worked in various oncology specialties, including urinary tract, genital system, breast, digestive system, skin, and lung cancers. The interviews lasted between forty minutes and an hour. They were conducted in the hospitals face-to-face, except two interviews which were conducted over the telephone due to COVID-19 restrictions. All interviews were conducted by a research assistant who is a graduate student in clinical psychology.

### 2.2. Research Tools

The survey questionnaire was written by the researchers and was validated using the content validation method by two physicians specializing in oncology and one specialist in public health and health policy. Following their comments, three ambiguous questions were clarified, and the questionnaire was piloted to examine reliability using Cronbach alpha (α = 0.73). The questionnaire included 15 statements describing perceptions and dilemmas regarding unfunded innovative treatments. Respondents were asked to rate their degree of agreement on a Likert scale ranging from 1 (strongly disagree) to 5 (strongly agree). The questionnaire also included socio-demographics such as gender, age, level of religiosity, country of birth, number of years of experience in oncology, whether the respondent works among the social periphery in Israel, and whether the respondent is currently, or once was, a member of the Basket Committee (Supplementary Materials Supp1). The statements in the questionnaire were grouped into several categories, as shown in Table 1.

The in-depth interviews were semistructured. The wording and order of the questions changed according to the interview dynamics to maintain continuity and flow and encourage openness among the interviewees (Supplementary Materials Supp1). 

### 2.3. Statistical Analysis

Data were analyzed using SPSS V. 26 (IBM Corp., Armonk, NY, USA). Correlations between the variables were examined using Pearson. Differences between groups were examined using t-tests for independent samples and one-way ANOVA, including post-hoc analysis using the Tukey method. Finally, a model of linear regression was constructed to predict perceptions toward recommending unfunded treatments. Based on Thiese et al. [13], and considering the limited sample size, a significance level of *p* < 0.10 was determined. The interviews were analyzed using a thematic analysis method in the ATLAS.ti software. The analysis included both deductive themes, arising from the research topic and literature review, and inductive themes that emerged from the data [14].

## 3. Results

### 3.1. Sample Characteristics and Perceptions toward Unfunded Treatments

Table 2 presents the characteristics of the sample of survey respondents. The mean age of the respondents was 57.06 ± 10.3. The mean number of years of work experience was 25.39 ± 9.9.

Table 3 presents the response distribution. Analysis of the survey population’s responses showed that 86% of respondents agreed that patients should be offered all treatment alternatives, including those that are not funded, regardless of the patient’s financial situation. Similarly, 88% indicated that physicians recommend innovative cancer treatments even if they are not funded. At the same time, 59% indicated that they often face a dilemma regarding whether to suggest a new unfunded therapy to patients with financial difficulties and without private health insurance. In addition, over a third of respondents (38%) stated that they felt embarrassed and uncomfortable discussing the cost of treatment with patients. 

### 3.2. Age and Gender Differences in Perceptions toward Unfunded Treatments

The respondents were divided into three age groups: under 50, 50–64, and 65 and over, to examine differences in perception between age groups regarding various aspects of innovative unfunded cancer treatments. A one-way ANOVA and post-hoc analysis using the Tukey method were performed. The findings are presented in Table 4.

As shown in Table 4, the youngest group of respondents, those up to the age of 50, were significantly more likely than the other two age groups to agree that when considering new treatments, physicians should consider the cost of treatment for both the patient and the health organization. Those aged 65 and over were significantly more likely to agree with the statement that patients prefer physicians to make decisions for them regarding treatment, regardless of its costs. Those between the ages of 50 and 64 were most likely to say they were aware of cases in which a patient’s condition worsened because they could not afford treatments. A significant difference was found between age groups regarding the social and ethical dilemmas of recommending innovative unfunded technologies. Those under 50 were more likely to agree more with the perception that the state pays an excessively high economic price for extensive treatment of cancer patients in terms of cost versus benefit. They were also more likely than the other groups to say that physicians should understand a patient’s socioeconomic status before recommending unfunded treatments, and that they often faced dilemmas regarding whether to recommend innovative care for patients with financial difficulties and without private health insurance. The younger generation of physicians seems to be more aware of the ethical and social dilemmas arising from the economic aspect of cancer treatment.

Significant differences by gender were found regarding the perception of the physician’s role in achieving compassionate care (t = 1.76, *p* < 0.10). Female physicians expressed a more positive perception than their male colleagues regarding physicians’ role in obtaining funding for innovative treatments for cancer patients who cannot afford them (mean 4.05 vs. 3.71, respectively).

### 3.3. Participant in National Basket Committee

To examine differences in perceptions between respondents who were involved in the treatment-rating process and those who did not participate in this process, t-tests for independent samples were performed. Significant differences were found regarding unfunded examinations and the perception of the physician’s role in achieving compassionate care. It emerged that those who participated in the Basket Committee were more likely to recommend unfunded examinations for their patients (t = −3.29, *p* < 0.001). Physicians who participated in the ranking process tended to see themselves as having more responsibility for delivering compassionate care to patients with financial difficulties. 

### 3.4. Associations between Perceptions

To examine the associations between the study variables, Pearson correlations were calculated. Analysis revealed that physicians who hold a positive perception toward the use of unfunded innovative examinations were also likely to say that oncologists should make an effort to achieve compassionate treatment for their patients (*p* < 0.01, r = 0.24), were more sensitive to inequality in healthcare (*p* < 0.001, r = 0.33), and felt responsible for considering the costs of innovative treatments (*p* < 0.05, r = 0.19). In addition, a negative correlation was found between having a positive perception regarding recommending new unfunded examinations to patients, and having social and ethical dilemmas regarding recommending innovative treatments (*p* < 0.05, r = −0.18). It was also found that physicians holding positive perceptions toward the use of innovative cancer treatments are likely to say that patients should be offered all treatment alternatives, including those that are not funded, regardless of their financial status (*p* < 0.05, r = 0.20), and to say that physicians should achieve compassionate care for their patients (*p* < 0.05, r = 0.17).

### 3.5. Prediction Model

Multiple linear regression was performed to examine the variables that predict whether physicians would recommend unfunded innovative cancer treatments. The following three statements from the survey were combined into one dependent variable using a mean calculation of the three statements (mean of the new variable: 3.75 ± 0.66): *(1) To the best of my knowledge, physicians tend to recommend new cancer treatments even if they are not funded by public healthcare; (2) Patients should be offered all treatment alternatives, including those that are not funded, regardless of their financial status;* and *(3) I often use examinations that are not funded by the healthcare system, for example, those using NGS technology*. 

A significant correlation was found between the new variable and four independent variables: inequalities in healthcare (*p* < 0.01, r = 0.27), the physician’s role in achieving compassionate care (*p* < 0.01, r = 0.26), social and ethical dilemmas involved in the recommendation of innovative technologies (*p* < 0.01, r = −0.25) and the physician’s responsibility to consider the cost of innovative cancer treatment (*p* < 0.05, r = −0.20).

Following this, a regression model was constructed. The model revealed that encountering patients whose medical condition worsened because they could not afford innovative treatments, and a perception that the physician should make an effort to find funding for expensive treatments for patients who cannot afford them predict the recommendation to use innovative unfunded cancer treatments. The percentage of explained variance is 15% (F = 5.22, R^2^ = 0.15, *p* < 0.001).

### 3.6. In-Depth Interviews

The interviews highlighted four themes: economic considerations in choosing treatment, physician–patient communication, the available publicly funded treatments, and discussion of treatment costs.

#### 3.6.1. Theme 1: Economic Considerations in Choosing a Treatment

Most interviewees agreed that all alternatives should be offered in any situation if it is done in a sensitive and considerate manner. Interviewees noted that it is not always possible to know their patients’ actual financial situation. Someone with limited financial resources may be able to get help from others. As Interviewee 7 explained: “*Experience shows that you cannot make assumptions. Some patients seem to have no resources, and then suddenly it turns out that someone is helping them, while there are very affluent patients who will not pay for unfunded treatments. Therefore, I do not make assumptions. I give the same information to all patients*”.

In contrast, Interviewee 11 argued that as long as there is no impairment in the quality of treatment, recommendations should be tailored to each patient’s financial situation: “*If patients do not have private insurance and have no money, there is a good chance that I would recommend the less expensive option...Just for that, a treatment that costs tens of thousands of shekels, family members can find that they lost their beloved family member and have huge debts in the end*”.

#### 3.6.2. Theme 2: Physician–Patient Communication

The characteristics of communication between physicians and patients vary depending on both parties’ nature and the perception of the physician’s role. Some doctors perceive that they should offer patients all of the options and let them choose. In contrast, others feel the need to guide patients in selecting treatments. As Interviewee 10 said: “*You have to present the picture. You have to tell the patient that this is a costly treatment, on the order of tens or hundreds of thousands of shekels, and make a recommendation. What does our boss say to patients? ‘I wouldn’t sell the car for it.’ Say it in words they can understand. You must see the patient sitting in front of you and understand his situation. Do not give patients hope when there is not. I do not think it is true that the doctor should simply present the options. One should always recommend what to do. It is called the ‘uncle test.’ What would you recommend that your uncle do?*”.

Interviewee 8 described a different approach: “*If we’re talking about unfunded treatments and a patient consult with me about what to do with the little money he has, I think it’s certainly our place to talk about what that means. Just as we help them know at what time to eat and whether to take medicine with food, we are also consulted on economic matters, and we must advise them to the best of our knowledge*”.

In contrast, Interviewee 14 focused solely on the medical aspect: “*My only consideration is the medical consideration. Many times, I suggest they get another opinion. Not only medicine costs money; sometimes, it is doing another imaging test that costs money. Or whether to go to a private surgeon who is better. There are situations when you can do either surgery or radiation. And sometimes it is easier for me to bring me another physician’s opinion if they want surgery. It makes my job easie*”.

#### 3.6.3. Theme 3: Publicly Funded Healthcare

A quarter of the interviewees had been members of the Health Fund Committee, either at the time of the survey or in the past. Interviewee 1 explained why economic considerations should not be part of the process: “*One of the questions we always ask, but for which there is no answer is: What is better, to give a less effective remedy to many people, or to give a highly effective remedy to fewer people? It is a cruel moral dilemma, and there is no answer. Therefore, in the discussion, you do not touch on costs*”.

All the interviewees praised the set of treatments available through the Israeli healthcare system in oncology, which provides adequate treatment solutions for many patients compared with other western countries. For example, interviewee 8 related: “*In Israel, the level of support that the public system gives is sufficient and is much better than what I saw compared to, for example, the United States or Europe. So, I am pleased that we can offer this as a healthcare system. I look at what we can give to patients, and we can offer a lot*”.

#### 3.6.4. Theme 4: Discussion of Treatment Costs

Two-thirds of the interviewed physicians said they become familiar with the costs of unfunded treatments before recommending them. Interviewee 16 addressed the ethical complexity that a discussion of treatment costs may create. In addition to the patient’s socioeconomic situation, there is a fear of being perceived as promoting certain drugs: “*When they ask me, and I’m speaking here about the range of costs, first, these things change. If I know the cost right now, it is unnecessary what the cost will be in three months. Second, I do not think I should talk to them [about this]. I do not deal in drugs. I can say yes, this medication costs such and such, but I am not part of this equation*”.

## 4. Discussion

The present study examined the ethical aspects of decisions regarding recommending and using unfunded innovative life-prolonging treatments. As seen in previous studies [15,16,17], most oncologists agreed that patients should be offered all treatment alternatives regardless of their financial situation. However, they often experience dilemmas regarding whether to recommend innovative treatments to patients with financial difficulties and without private health insurance. Such dilemmas in patient–physician discussions about cost were also mentioned in past studies [18,19,20]. Additionally, over a third of the participants indicated that they feel embarrassed and uncomfortable discussing the cost of care with patients—another trend indicated in recent studies [21,22]. 

In-depth interviews revealed a complex picture. Some physicians, especially the older and more experienced, said that they first try to ascertain the patients’ financial status and whether they have private healthcare insurance. However, most physicians said that they are careful to suggest all treatment alternatives because some patients do manage to raise funds from family members or a crowdfunding campaign. Moreover, some oncologists have personally approached pharmaceutical companies to ask for assistance in providing compassionate care or referred patients to various associations for help. However, the dilemma of conscience that is involved in presenting all options to a patient who is unlikely to be able to afford them arose in all interviews, despite the Israeli Ministry of Health’s guidelines that physicians should offer all relevant treatment alternatives regardless of the patient’s socioeconomic status, which is intended to free physicians from this ethical dilemma and pass the decision to the patient. 

A survey of physicians from the United States and Canada found that, despite significant differences between the two countries’ healthcare systems, most physicians agreed that spending large sums of money on treatments might affect patients’ course of treatment. However, more than half of the surveyed physicians held no discussions regarding their patients’ financial condition due to their discomfort when they tried to initiate such a discussion [23]. Similarly, in the current study, some physicians said they felt a responsibility to conduct a conversation that also concerned the economic implications of the treatment, while others thought that they should discuss only clinical issues. Similarly, Altomare et al. [10] found that 90% of oncologists always offer their patients all treatment options regardless of cost, and about half of them said they have never performed cost-based prioritization. In addition, most oncologists agreed that it was their responsibility to consider the personal and social costs of innovative cancer treatments. Still, over 70% indicated that they did not have the resources, knowledge, or ability to discuss treatment costs with their patients.

In the current study, physicians aged 65 and over were more likely to agree with the idea that patients prefer that physicians make decisions for them regardless of their costs. The younger generation of physicians seems to be more aware of the ethical and social dilemmas that arise from the financial costs of cancer treatments. Most of the interviewed physicians stated that they consider themselves responsible for choosing the most appropriate treatment for the patient, who expects this from the physician. Previous studies have already noted the prevalence of the tendency to give a physician full authority over treatment decisions [24,25]. It can be assumed that the gaps between the various age cohorts of physicians are related to broad processes that have changed the face of medicine, shifting from a paternalistic approach, according to which the physician knows what is best for the patient and should not be challenged, and moving to a liberal approach, which holds that patients are the best arbiters of their condition and should be placed at the center of decision making [26,27,28]. Despite the existence of various approaches and positions regarding treatment decisions, the perception that the physician should make decisions regarding treatment is still dominant. However, when it comes to terminal illnesses, it is possible that, despite the remnants of this paternalistic culture, physicians are in a more rational, less emotional position than are their patients, and this enables them to make a more “rational” cost-benefit analysis and thus make better decisions for their patients.

The current study found that being aware of patients whose medical condition worsened significantly because they could not afford unfunded treatments, alongside a perception that physicians should make an effort to help find funding for treatments that are not covered by public healthcare, predicted a recommendation to use unfunded innovative cancer treatments. In addition, about one-third of the respondents said they had encountered situations in which financial difficulties directly affected the patient’s medical condition [29]. This finding, combined with positive experiences in achieving compassionate care, contributes to the fact that these physicians have expressed a greater tendency to recommend the use of unfunded drugs in practice.

A similar survey conducted in the United States found a gap between physicians’ knowledge of experimental treatments and their willingness to assist patients in obtaining these treatments [30]. Awareness of cases in which patients are directly affected by a lack of access to experimental treatments may be what bridges the gap between clinical knowledge and willingness to help. In addition, the interviews indicated that encountering cases in which an economic situation led to a worsening of the medical condition also increased the physicians’ motivation to help find funding for treatments.

## 5. Study Limitations

The present study has several limitations. First, the survey was conducted among only 127 oncologists. At the same time, the response rate to the survey was high and included about 50% of all oncologists registered with the Israeli Society for Clinical Oncology and Radiation Therapy. Another limitation is that a questionnaire written by the researchers, and not previously tested in other research frameworks, was used because few studies have been conducted on this topic. Nevertheless, the questionnaire is valid and high reliability of the questionnaire items was found.

## 6. Conclusions

The present study sheds light on some of the ethical dilemmas faced by oncologists in the decision-making process regarding treatments not funded by the public healthcare system. Physicians feel an ethical and professional commitment to offer patients the best medical care, and at the same time feel a moral obligation to discuss treatment costs and minimize the financial difficulties for patients and their families that may be anticipated as a result of using innovative treatments, which may prolong the patient’s life but do not offer a cure. The findings highlight the fact that, although the Israeli Ministry of Health guides physicians to offer all alternatives, in practice physicians meet the difficulties of their patients, face the ethical vs. clinical dilemma, and try to balance between the two while considering the patient’s character and needs. The tensions presented in our study cannot be solved solely within the physician–patient dyad, they must involve structural considerations relevant to the specific country’s context. Within the Israeli context, there is an ongoing pressure on the Ministry of Finance to automatically update the health basket according to demographic growth and technology indices. This would allow more evidence-based, funded treatments to be added to the basic health basket, in addition to their availability via private insurance, in order to reduce inequities among patients and to reduce the ethical and emotional burden on physicians.

There is a need for an open public discussion of these issues in focus groups that include physicians, patients, and experts in bioethics. This need becomes even more pronounced when it is apparent that most oncologists in Israel recommend unfunded treatments to patients and even try to obtain funding for their patients. Therefore, it is necessary to develop guidelines for oncologists to help them conduct comprehensive and in-depth discussions with patients regarding all aspects of cancer treatment and to make a joint decision with patients regarding the optimal treatment for their illness. Further research in the field can focus on the psychosocial aspect of the physician–patient relationship, communication styles, inclusivity, and empathy between the parties.

## Figures and Tables

**Table 1:** Research variable means and standard deviations.

Type	Issues and Positions	Number of Statements	Mean ± S.D.
Dependent variables	Recommending innovative unfunded treatments to patients	1	4.41 ± 0.89
	Actual use of innovative unfunded treatments	1	4.07 ± 0.83
	Use of innovative unfunded tests	1	2.74 ± 1.18
Independent variables	Perception of the physician’s role in achieving compassionate care	1	3.88 ± 1.08
	Need for physician–patient discussion about costs and benefits of innovative cancer treatments	3	3.76 ± 0.90
	Responsibility for choosing the treatment	1	3.45 ± 1.11
	Social and ethical dilemmas associated with recommending innovative cancer treatments	3	3.14 ± 0.92
	Preference for clinical efficacy of treatment for the patient	1	3.00 ± 1.07
	Physician’s responsibility for considering costs of innovative cancer treatments	2	2.81 ± 1.03

**Table 2:** Characteristics of survey sample.

Variable		*n*	%
Gender	Male	63	50%
	Female	63	50%
Country of birth	Israel	67	53%
Country of medical education	Israel	64	52%
Works in the social periphery	Yes	61	49%
Participant in National Basket Committee	Yes	86	69%

**Table 3:** Survey response distribution.

Statements	Agree	Neutral	Disagree
The patient should be offered all treatment alternatives, including those that are not funded, regardless of their financial situation	86%	8%	6%
I feel ready to discuss with the patient the cost-benefit of a treatment I recommend.	88%	6%	7%
To the best of my knowledge, physicians tend to recommend cancer treatments even if they are not funded by public healthcare.	78%	19%	3%
Discussion of financial costs of healthcare may harm the physician–patient relationship.	11%	18%	71%
A physician can make an effort to find funding for unfunded treatments for patients who cannot afford them.	64%	26%	10%
When considering a new treatment, physicians should consider the cost of treatment for the patient.	65%	16%	19%
In my opinion, patients prefer that the physician decides on the recommended treatment of the disease, regardless of cost.	52%	29%	19%
I have often faced the dilemma of whether to recommend innovative care for patients with financial difficulties (without private health insurance).	59%	15%	26%
Patients always prefer clinical efficacy, regardless of the price they will have to pay for the treatment	26%	48%	27%
Physicians should learn a patient’s socioeconomic status before making a recommendation for unfunded treatment.	44%	18%	38%
It is embarrassing/uncomfortable for me to discuss the cost of treatments for which they must pay with a patient.	38%	23%	39%
I am aware of patients whose medical condition significantly worsened because they could not afford unfunded treatments.	35%	18%	47%
The state pays the economic price for extensive treatments for cancer patients regarding cost versus benefit.	29%	26%	46%
I often use tests not funded by the healthcare system, such as those using NGS technology.	25%	31%	44%
When considering new treatment, physicians should consider the cost of treatment for the HMO.	12%	19%	69%

**Table 4:** Analysis of variance for comparisons between age groups regarding the study variables.

Variable	Age Group	Mean	SD	F
Physician’s responsibility to consider costs of innovative cancer treatments	Under 50	3.39	0.90	7.272 ***
	50–64	2.65	0.92	
	65 and over	2.56	0.88	
Responsibility for choosing the treatment	Under 50	2.96	1.13	4.115 **
	50–64	3.49	1.12	
	65 and over	3.80	0.91	
Inequalities in healthcare	Under 50	2.26	1.23	4.035 **
	50–64	3.15	1.37	
	65 and over	2.46	1.45	
Social and ethical dilemmas associated with recommending innovative cancer treatments	Under 50	3.51	0.80	3.127 **
	50–64	3.15	0.93	
	65 and over	2.91	0.88	
Preference for clinical efficacy of treatment for the patient	Under 50	2.88	0.95	2.783 *
	50–64	2.90	1.14	
	65 and over	3.50	1.21	
Use of innovative unfunded treatments	Under 50	4.15	0.60	2.710 *
	50-64	4.12	0.84	
	65 and over	3.96	0.97	
Need for physician–patient discussion about costs and benefits of innovative cancer treatments	Under 50	3.58	0.76	1.538
	50–64	3.93	0.88	
	65 and over	3.88	0.82	
Use of innovative unfunded tests	Under 50	2.41	1.25	1.334
	50–64	2.87	1.32	
	65 and over	2.52	0.96	
Recommendation for unfunded innovative treatments	Under 50	4.12	0.82	1.011
	50–64	4.45	0.99	
	65 and over	4.37	1.01	
Perception of the physician’s role in achieving compassionate care	Under 50	3.65	1.13	0.187
	50–64	3.83	1.09	
	65 and over	3.77	1.24	

* *p* < 0.10, ** *p* < 0.05, *** *p* < 0.01.

## Data Availability

The data that support the findings of this study are available from the corresponding author.

## Supplementary Materials

The following are available online at https://www.mdpi.com/article/10.3390/curroncol28040254/s1. The research questionnaire and interview guide are included in Supp1.
