# Supplementary material for: Recommending Unfunded Innovative Cancer Therapies: Ethical vs. Clinical Perspectives among Oncologists on a Public Healthcare System—A Mixed-Methods Study
Source: Curr Oncol. 2021 Aug 2;28(4):2902–13. doi: 10.3390/curroncol28040254 (PMC8395438; doi:10.3390/curroncol28040254)
Supplement: Supplementary file 1 [file curroncol-28-00254-s001.zip › curroncol-1308984-supplementary.pdf]

Article

Recommending Unfunded Innovative Cancer Therapies: Ethical vs. Clinical Perspectives among Oncologists on a Public Healthcare System—A Mixed-Methods Study

Osnat Bashkin [1,*,†], Keren Dopelt [1,2,†], Noam Asna [3] and Nadav Davidovitch [2]

Supplementary Materials

1. The Questionnaire for Oncologists

Greetings,

Oncologists face many dilemmas regarding recommending unfunded treatments. This questionnaire is administered as part of a study examining these dilemmas. Responding to this questionnaire is voluntary. The survey is anonymous, and the answers will remain confidential. Some statements are written in the masculine tense for convenience but are intended to refer to males and females. We would appreciate it if you would complete the attached questionnaire. It is expected to take about five minutes to complete.

For further information, contact Dr. Osnat Bashkin via email: obashkin@gmail.com

Circle the number indicating the extent to which you agree with the following statements:

	Strongly Disagree				Strongly Agree
1. To the best of my knowledge, physicians tend to recommend cancer treatments even if they are not funded by public healthcare.	1	2	3	4	5
2. Physicians should learn a patient's socioeconomic status before making a recommendation for unfunded treatment.	1	2	3	4	5
3. I have often faced the dilemma of whether to recommend innovative care for patients with financial difficulties (without private health insurance).	1	2	3	4	5
4. Patients always prefer clinical efficacy, regardless of the price they will have to pay for the treatment.	1	2	3	4	5
5. When considering an innovative treatment, physicians should consider the cost of treatment <u>for the patient.</u>	1	2	3	4	5
6. When considering innovative treatment, physicians should consider the cost of treatment <u>for the HMO</u>.	1	2	3	4	5
7. I feel ready to discuss with the patient the cost-benefit of a treatment I recommend.	1	2	3	4	5
8. The patient should be offered all treatment alternatives, including those that are not funded, regardless of his financial situation	1	2	3	4	5
9. It is embarrassing/uncomfortable for me to discuss the cost of treatments for which he must pay with a patient.	1	2	3	4	5
10. Discussion of financial costs of healthcare may harm the physician-patient relationship.	1	2	3	4	5
11. The state bears the economic price for extensive treatments for cancer patients regarding cost versus benefit.	1	2	3	4	5
12. I often use tests not funded by the healthcare system, such as those using NGS technology.	1	2	3	4	5
13. A physician can make an effort to find funding for unfunded treatments for patients who cannot afford them.	1	2	3	4	5
14. I am aware of patients whose medical condition worsened because they could not afford unfunded treatments.	1	2	3	4	5
15. In my opinion, patients prefer that the physician decides on the recommended treatment of the disease, regardless of costs.	1	2	3	4	5

Finally, please answer a few general questions for research purposes:

Gender: 1. Male 2. Female

2. Age: _______

3. Religion: 1. Jewish 2. Muslim 3. Christian 4. Atheist 5. Other: ___

4. Country of birth 1. Israel 2. Former USSR 3. Other: ______________

5. At which university and in which country did you study medicine? ______________________________

6. Number of years of experience as an oncologist (including residency): ________

7. Do you work in the social periphery? 1. Yes 2. No 3. Yes, in the past

8. Were you involved in the oncology treatment-rating processes for the public healthcare system?

1. Yes 2. No

9. If you were to write new guidelines for physicians <u>not limited to oncologists</u> regarding cases in which the patient does <u>not</u> have private health insurance, do you think physicians should offer all alternatives, even the most expensive ones, or consider the patient's financial situation?

1. All alternatives should be suggested regardless of the patient's financial situation

2. The patient's financial situation should be taken into account, and only publicly funded alternatives should be offered

3. Other: ______________________

2. Guide for Interviews with Oncologists

1. Tell me a little about yourself. How long have you worked in the oncology department? Why did you choose this specialization?
2. Can you estimate how many patients with advanced stages of the disease you treat per week?
3. Have you been involved in the Health Fund Committee processes for rating oncology treatments funded by the public healthcare system?
4. What do you know about the processes of testing innovative treatments in the field of oncology to include them in the set of funded treatments before discussion by the Health Fund Committee?
5. Do you often use tests such as NGS technology that are not funded by the public healthcare system in Israel?
6. Have you ever had to recommend one of your patients to take out a loan or sell the property to fund oncology treatments, or have you heard of such a case from another oncologist? If so, what is your position on the matter?
7. Have you experienced an extraordinary and unexpected success when using expensive, privately funded treatments for one of your patients?
8. Have you ever experienced clinical failure when using treatments that are considered "life-saving"?
9. Do you hold discussions on the issue of spending large sums of money for oncology treatments with the patient alone, only with the patient's family members, or with everyone together?

10. The development of cancer treatments is gaining momentum, and some of the treatments are not publicly funded. Are you aware of the costs of such treatments? Do you have a way of knowing the costs?
11. If there is a less expensive alternative to treatment with the same benefit, do you think the physician should recommend the less expensive choice or present both to the patient?
12. Do you offer your patients all treatment alternatives, including those not funded, regardless of their financial situation?
13. Can you share a dilemma you have had concerning a case in which you believed that treatment could prolong a patient's life but that the patient would have difficulty financing it? What did you do in this situation? Did you try to find ways to get funding?
14. Do you think it is your task to know and discuss the costs of the treatments? Or should you only offer the clinical alternatives that seem most appropriate to you?
15. Do you think society pays an excessively high economic price for extensive treatments for cancer patients in terms of cost versus benefit and financial resources that could have been invested elsewhere?
16. Suppose you were to write guidelines for physicians, not necessarily oncologists. For example, would you suggest that physicians offer patients all alternatives, even the most expensive ones, or consider the patient's financial situation?
